# Stomatognathic Diseases Reveal Bidirectional Link Between Diabetes Mellitus and Coronary Artery Calcium: A Cross‐Sectional Study Using Multi‐Way Array Analysis

**DOI:** 10.1002/hsr2.71280

**Published:** 2025-09-29

**Authors:** Tuan D. Pham

**Affiliations:** ^1^ Barts and The London School of Medicine and Dentistry Queen Mary University of London London UK

**Keywords:** artificial intelligence, coronary artery calcification, patient characteristics, stomatognathic diseases, tensor decomposition

## Abstract

**Background and Aims:**

Understanding the relationship between diabetes mellitus and cardiovascular risk is crucial for effective healthcare. Diabetes mellitus (DM), a complex metabolic disorder, is closely linked to an increased risk of cardiovascular diseases. Factors such as endothelial dysfunction, inflammation, and metabolic disturbances contribute to this heightened risk. Gaining insights into this relationship can help healthcare professionals provide timely and personalized care. This study explores the bidirectional relationship between DM and coronary artery calcification (CAC), mediated by stomatognathic diseases, using advanced data science techniques.

**Methods:**

This study uses a publicly available data set of 212 patients from Dutch hospitals to explore the connections among patient characteristics, stomatognathic diseases, and CAC score. Tensor decomposition techniques were employed to investigate the relationship between DM and CAC. Patient characteristics and dental conditions were integrated into tensor models for three groups: without DM, with DM, and with CAC. Additionally, nonlinear dynamics, visual analyses, and machine learning enriched the investigation.

**Results:**

Tensor decomposition revealed patterns across the three categories, incorporating patient characteristics and dental conditions. The k‐NN (nearest neighbor) search examined similarities among tensor coefficients, highlighting a bidirectional link between DM and CAC. Fuzzy recurrence plots and entropy measures quantified distinctive patterns among subjects without DM, with DM, and with CAC.

**Conclusion:**

The reciprocal interaction between DM and CAC tertiles 2 and 3 emphasizes the need for a broader analytical perspective. Incorporating patient characteristics and dental health in the analysis uncovers latent patterns, providing insights. Oral conditions emerge as key indicators, offering a detailed view of the complex relationship between DM and CAC.

## 1 Introduction

In the landscape of contemporary healthcare, understanding the intricate interrelationships between various health parameters is imperative for comprehensive patient care. A particularly intriguing connection lies in the bidirectional relationship between diabetes mellitus (DM) and cardiovascular risk, two conditions of global health significance [1, 2].

Cardiovascular disease continues to be the leading cause of mortality and disability among individuals with DM that worsens the underlying mechanisms involved in atherosclerosis and heart failure [3]. However, many therapeutic approaches primarily targeting glycemic control with current medications or interventions do not adequately address these mechanisms. Individuals with diabetes still experience higher rates of cardiovascular complications compared to those without the condition [3].

Strong association between DM and cardiovascular disease has been reported in the literature [4]. Common cardiovascular risk factors like obesity, hypertension, and dyslipidemia are prevalent in diabetic patients, elevating their susceptibility to cardiac events. Hence, it is suggested that it is imperative to target cardiovascular risk factors in patients with DM to mitigate the long‐term cardiovascular complications associated with the disease [4].

The bidirectional interplay between diabetes and cardiovascular risk is far from linear, as evidenced by the multi‐faceted nature of their interactions [5]. Recognizing the potential role of dental conditions as pivotal contributors to the complex relationship of diseases can open up new dimensions in human understanding of the pathophysiological mechanisms at play [6, 7, 8].

In particular, oral health is increasingly recognized as an integral component of systemic health, with accumulating evidence supporting its connection to both metabolic and cardiovascular diseases [9]. Among oral conditions, periodontitis and related stomatognathic diseases have been strongly linked to DM and cardiovascular risk [10, 11, 12]. Periodontal disease and diabetes share a bidirectional relationship, whereby poor glycaemic control exacerbates oral inflammation and infection, while periodontal pathogens may worsen insulin resistance and systemic inflammation [13, 14].

Furthermore, a growing body of epidemiological and mechanistic research supports the association between periodontitis and atherosclerotic cardiovascular disease [15, 16]. These findings suggest that oral health conditions may serve not only as clinical indicators of systemic disease but also as modifiable risk factors with potential implications for prevention and intervention.

Despite this, much of the existing literature relies on traditional statistical approaches, which may not fully capture the complex, multidimensional interactions among oral pathology, diabetes, and cardiovascular outcomes. Advanced computational methods are now being explored to reveal latent patterns and interdependencies that elude linear modeling techniques [17, 18].

This study employs a cutting‐edge approach in data science, utilizing multi‐way array analysis with tensor decomposition [19, 20] to comprehensively investigate the joint influence of patient characteristics and dental health conditions in relation to diabetes and coronary artery calcification (CAC). The methodology addressed in this study allows for an analytical exploration of the bidirectional link, acknowledging the intricate dynamics between diabetes and cardiovascular risk within a multi‐dimensional framework. The objective is not merely to reaffirm the established association between diabetes and cardiovascular risk but to explore deeper into their reciprocal influence. The dental context adds granularity to the analysis, recognizing oral health as an integral component in the broader spectrum of systemic well‐being.

This study aims to investigate the bidirectional relationship between DM and CAC through the presence of stomatognathic diseases, using a multi‐way analytical approach that combines tensor decomposition with fuzzy recurrence analysis to uncover latent interdependencies in multidimensional clinical data.

## 2 Methods

### 2.1 Data Set

This study used a publicly available data set [21], featuring a cohort of 212 patients obtained from three Dutch hospitals, of which 114 participants were male. The average age of the cohort was at 57.8 years. Within this diverse group, 32 individuals presented with diabetes, 85 with hypercholesterolemia, and 128 were undergoing treatment for hypertension. The smoking history of the cohort was classified into three categories: 86 were non‐smokers, 86 were former smokers, and 39 were current smokers.

Patient characteristics consist of sex, age, smoking status, diabetes mellitus, hypercholesterolemia, hypertension, and body mass index (BMI). Stomatognathic diseases encompass conditions such as missing teeth, dental implants, alveolar bone loss, dental caries, endodontically treated teeth, periapical radiolucencies, peri‐implant bone loss, impacted teeth, and odontogenic cysts. CAC is evaluated using CAC scores, typically categorized into tertiles to stratify cardiovascular risk.

### 2.2 Multi‐Way Array Analysis

The general representation of the elements within an n‐way tensor is expressed mathematically as [22, 23].

(1) $$h_{\mathrm{ij}\text{…}n}\approx \sum_{f=1}^{F}a_{\mathrm{if}}\;b_{\mathrm{jf}}\;\cdots \;c_{\mathrm{nf}},$$

where F represents the number of factors, $h_{\mathrm{ij}\text{…}n}$ are the elements of a tensor, and $a_{\mathrm{if}}$, i=1,…,I, $b_{\mathrm{jf}}$, j=1,…,J, and $c_{\mathrm{nf}}$, n=1,…,N, are elements of the loading matrices A, B, and C, respectively.

A tensor, denoted as $\underline{H}$, can be expressed as

(2) $$\underline{H}\approx \sum_{f=1}^{F}a_{f}⊗b_{f}⊗\cdots ⊗c_{f},$$

where ⊗ denotes the outer product, and $a_{f}$, $b_{f}$, and $c_{f}$ are the f‐column vectors of loading matrices A, B, and C, respectively. The computation of the matrices A, B, and C can be achieved through the application of the PARAFAC (parallel factor analysis) decomposition model [24, 25].

Consider a 3‐way array with loading matrices A, B, and C. The PARAFAC decomposition can be achieved by utilizing the method of alternating least squares to minimize the error of approximation within the model: H=a(b⊗c), where the unfolded array H=I×JN. For scenarios involving more than one factor, let b⊗c=Z. The expression for $\underline{H}$ can then be reformulated as:

(3) H=AZ.

If Z is given by initializing b and c, the estimation of A becomes:

(4) $$A=HZ^{T}{\left(ZZ^{T}\right)}^{-1}.$$

Similar procedures are employed to estimate B and C. For B, the tensor $\underline{H}$ is unfolded into the matrix H of size J×IN, and Z is derived from A and C. Similarly, for estimating C, H of size N×IJ, and Z is determined from A and B.

To gain insights into information provided about patient characteristics and stomatognathic diseases across three scenarios—1) diabetes, 2) no diabetes, and 3) CAC—distinct 2‐way and 3‐way tensor models are formulated as follows.

For a 2‐way tensor focused on patient characteristics in the absence of diabetes, denoted as ${\underline{G}}_{\mathrm{nDM}-C}$:

(5) $${\underline{G}}_{\mathrm{nDM}-C}=S_{\mathrm{nDM}}\times C_{\mathrm{nDM}},$$

where $S_{\mathrm{nDM}}$ represents the number of subjects without diabetes, and $C_{\mathrm{nDM}}$ includes the clinical data of patients without diabetes.

For a 2‐way tensor focusing on patient characteristics in the presence of diabetes, denoted as ${\underline{G}}_{\mathrm{DM}-C}$:

(6) $${\underline{G}}_{\mathrm{DM}-C}=S_{\mathrm{DM}}\times C_{\mathrm{DM}},$$

where $S_{\mathrm{DM}}$ denotes the number of subjects with diabetes, and $C_{\mathrm{DM}}$ is the clinical data of patients with diabetes.

For a 2‐way tensor addressing patient characteristics related to CAC, denoted as ${\underline{G}}_{\mathrm{CAC}-C}$:

(7) $${\underline{G}}_{\mathrm{CAC}-C}=S_{\mathrm{CAC}}\times C_{\mathrm{CAC}},$$

where $S_{\mathrm{CAC}}$ reflects the number of subjects with CAC tertiles 2 or 3, and $C_{\mathrm{CAC}}$ consists of the clinical data of patients with CAC tertiles 2 or 3.

For a 2‐way tensor examining stomatognathic diseases in the absence of diabetes, denoted as ${\underline{G}}_{\mathrm{nDM}-D}$:

(8) $${\underline{G}}_{\mathrm{nDM}-D}=S_{\mathrm{nDM}}\times D_{\mathrm{nDM}},$$

where $S_{\mathrm{nDM}}$ denotes the number of subjects without diabetes, and $D_{\mathrm{nDM}}$ is the stomatognathic disease data of patients without diabetes.

For a 2‐way tensor investigating stomatognathic diseases in the presence of diabetes, denoted as ${\underline{G}}_{\mathrm{DM}-D}$:

(9) $${\underline{G}}_{\mathrm{DM}-D}=S_{\mathrm{DM}}\times D_{\mathrm{DM}},$$

where $S_{\mathrm{DM}}$ represents the number of subjects with diabetes, and $D_{\mathrm{DM}}$ comprises the stomatognathic disease data of patients with diabetes.

For a 2‐way tensor addressing stomatognathic diseases related to CAC, denoted as ${\underline{G}}_{\mathrm{CAC}-D}$:

(10) $${\underline{G}}_{\mathrm{CAC}-D}=S_{\mathrm{CAC}}\times D_{\mathrm{CAC}},$$

where $S_{\mathrm{CAC}-D}$ is the number of subjects with CAC tertiles 2 or 3, and $D_{\mathrm{CAC}}$ consists of the stomatognathic disease data of patients with CAC tertiles 2 or 3.

To examine the combined impact of patient characteristics and stomatognathic diseases in three distinct scenarios—1) no diabetes, 2) diabetes, and 3) CAC—a series of 3‐way tensor models are formulated as follows.

For a 3‐way tensor analyzing both patient characteristics and stomatognathic diseases in the absence of diabetes, denoted as ${\underline{G}}_{\mathrm{nDM}-C-D}$:

(11) $${\underline{G}}_{\mathrm{nDM}-C-D}=S_{\mathrm{nDM}}\times C_{\mathrm{nDM}}\times D_{\mathrm{nDM}}.$$

Similarly, for a 3‐way tensor exploring both patient characteristics and stomatognathic diseases in the context of diabetes, denoted as ${\underline{G}}_{\mathrm{DM}-C-P}$:

(12) $${\underline{G}}_{\mathrm{DM}-C-D}=S_{\mathrm{DM}}\times C_{\mathrm{DM}}\times D_{\mathrm{DM}}.$$

Likewise, for a 3‐way tensor investigating both patient characteristics and dental stomatognathic diseases related to CAC, denoted as ${\underline{G}}_{\mathrm{CAC}-C-D}$:

(13) $${\underline{G}}_{\mathrm{CAC}-C-D}=S_{\mathrm{CAC}}\times C_{\mathrm{CAC}}\times D_{\mathrm{CAC}}.$$

### 2.3 Method of k‐Nearest Neighbor Search

To assess the similarity among three categories—diabetes, no diabetes, and CAC tertiles 2 and 3—the k‐nearest neighbor (k‐NN) search [26] appears instrumental and is adopted in this study. This method involves categorizing query points based on their proximity to points in a training data set, offering a straightforward yet effective classification approach. Various metrics exist for measuring distance, and here, the K‐d tree (K‐dimensional tree) technique [27], employing Euclidean distance, is employed for the k‐NN search.

### 2.4 Fuzzy Recurrence Plots

The FARAFAC decompositions resulted from different tensor models can be visually examined by means of the fuzzy recurrence plot (FRP) method [28]. Mathematically, an FRP for a PARAFAC decomposition factor, denoted as u, is described as follows: Let $u=(u_{1},u_{2},\ldots ,u_{T})$, where T is the number of subjects included in the tensor model. Let m and τ denote the embedding dimension and time delay for FRP construction, respectively. The phase space of u, denoted as X, is expressed as

(14) $$X=(x_{1},x_{2},\ldots ,x_{L}),$$

where L=T−(m−1)τ, and its elements are formed by

(15) $$x_{i}=(u_{i},u_{i+\tau },\ldots ,u_{i+(m-1)\tau }),i=1,\ldots ,L.$$

Utilizing the phase space and the fuzzy c‐means (FCM) algorithm [29], an FRP is computed as follows. Given a number of clusters c, the FCM partitions X into c clusters: $\left(v_{1},v_{2},\ldots ,v_{c}\right)$. The FCM also associates each element of X with real membership grades in [0, 1], expressing degrees of similarity between each element and all clusters, denoted as $\mu (x_{i},v_{k})$, i=1,…,L, k=1,…,c. A higher membership value indicates a greater degree of recurrence in the phase space. As a result, an FRP, denoted as F, is formulated as

(16) $$F(i,j)=\mu (x_{i},x_{j}),i,j=1,\ldots ,L,$$

where $\mu (x_{i},x_{j})$ represents the degree of similarity between two elements $x_{i}$ and $x_{j}$. This similarity can be inferred based on fuzzy similarity relations [30] according to the rules of self‐similarity, symmetry, and transitivity [28].

The normalized entropy of an FRP, denoted by E, quantifies the distribution of image intensity within the FRP and is defined as follows [31]:

(17) $$E=\frac{1}{L\times L}\sum_{i=}^{L}\sum_{j=1}^{L}-\mu (x_{i},x_{j})\;\log_{2}\mu (x_{i},x_{j})-[1-\mu (x_{i},x_{j})]\;\log_{2}[1-\mu (x_{i},x_{j})].$$

The above expression indicates that a lower entropy value corresponds to a more certain degree of either recurrent (darker pixels) or non‐recurrent (brighter pixels) events within the FRP.

## 3 Results

To facilitate the computation of tensor decomposition and enhance the analytical capabilities of the data set, categorical variables underwent conversion into numerical representations. The transformation involved assigning numerical values to categorical responses, where “Never” was encoded as 1, “Ever” as 2, “Present” as 1, “No” as 0, and “Yes” as 1. Additionally, the male subject with an indeterminate smoking status marked as “Unknown” was excluded from the data set to maintain data integrity. To ensure the precision of subsequent analyses, variables such as BMI and bone loss, characterized by numerous missing values, were deliberately omitted. This meticulous curation aimed at optimizing the data set for robust tensor decomposition and subsequent analyses.

**Figure 1:**
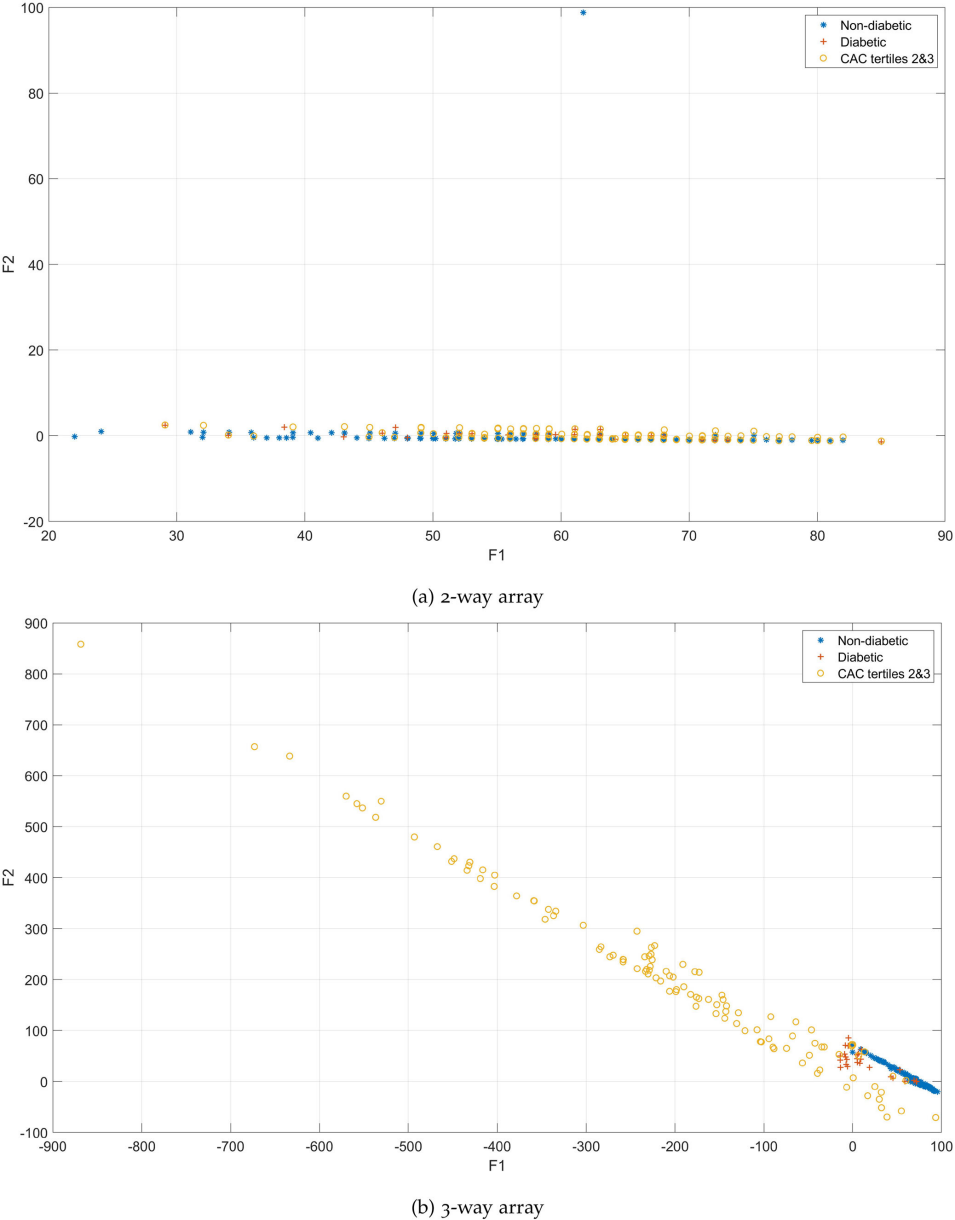
PARAFAC decomposition factors with the inclusion of (a) patient characteristics, and (b) patient characteristics and stomatognathic diseases. (a) 2‐way array, (b) 3‐way array.

Figure 1 shows a visual representation, unveiling the details of the PARAFAC tensor decomposition factors $F_{1}$ and $F_{2}$. These factors are extracted from both 2‐way (subjects × patient characteristics) and 3‐way (subjects × patient characteristics × stomatognathic diseases) arrays, analyzed across subjects without DM, with DM, and with CAC. This multi‐way array analysis not only identifies patterns within the data but also highlights the nuanced relationships of the variables existing in the data set.

Building upon the findings from Figure 1, a data‐driven exploration unfolds through a k‐NN search with k = 1, aimed at exploring deeper into the correlation between DM and CAC. The findings resulting from this investigation are presented in Tables 1 and 2, providing an overview of the correlations within both 2‐way and 3‐way array models.

**Table 1:** k‐NN search on 2‐way array signatures of patient characteristics.

Class	Count	Percentage
Finding diabetes closest to:		
CAC	22	68.75
No diabetes	10	31.25
Finding CAC tertiles 2 & 3 closest to:		
Diabetes	37	26.24
No diabetes	104	73.76

**Table 2:** k‐NN search on 3‐way array signatures of patient characteristics and stomatognathic diseases.

Class	Count	Percentage
Finding diabetes closest to:		
CAC	20	62.50
No diabetes	12	37.50
Finding CAC tertiles 2 & 3 closest to:		
Diabetes	103	73.05
No diabetes	38	26.95

**Figure 2:**
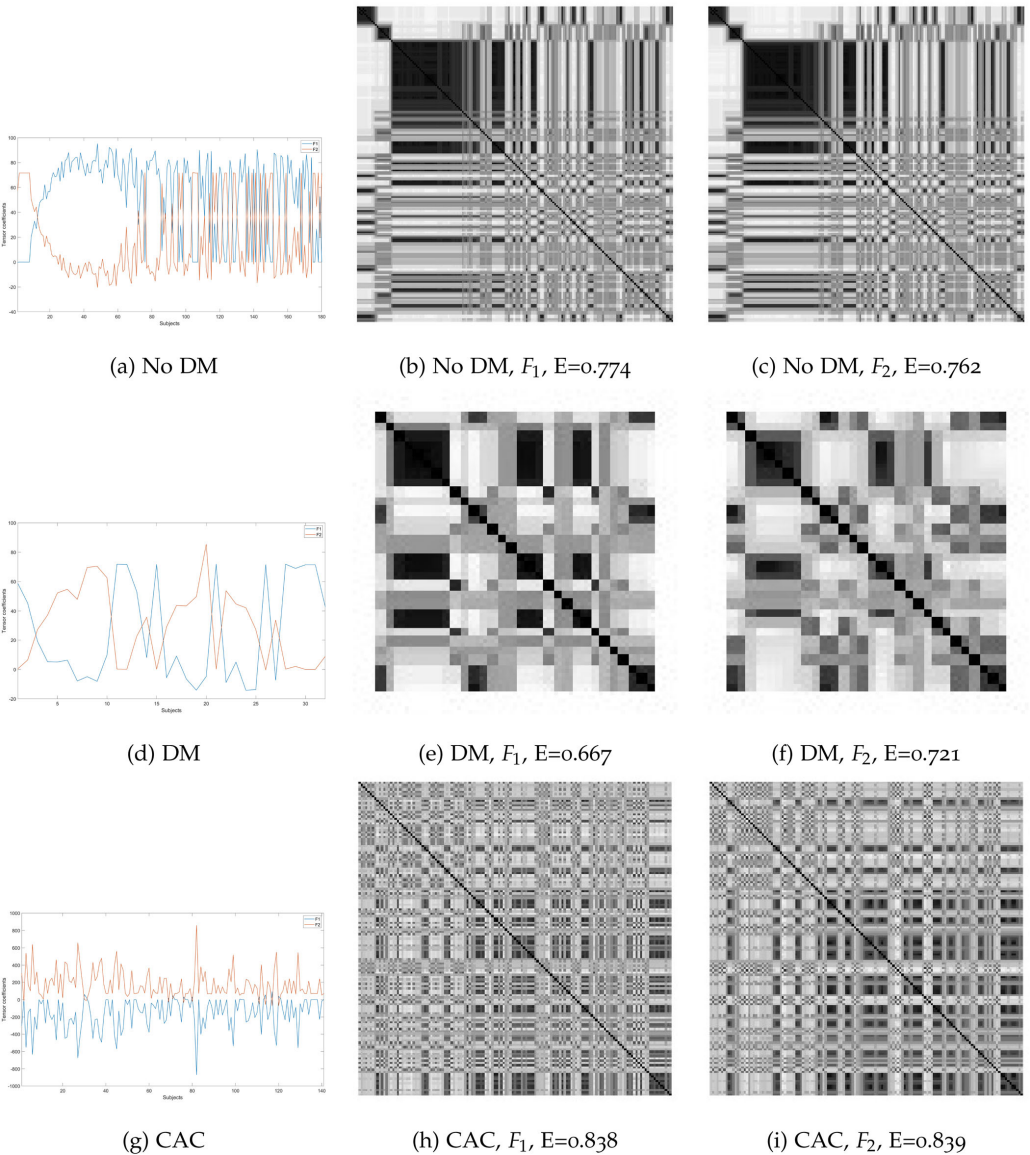
Coefficients of PARAFAC decomposition factors $F_{1}$ and $F_{2}$, and their FRPs computed from 3‐way tensor models. (a) No DM, (b) No DM, $F_{1}$, E = 0.774, (c) No DM, $F_{2}$, E = 0.762, (d) DM, (e) DM, $F_{1}$, E = 0.667, (f) DM, $F_{2}$, E = 0.721, (g) CAC, (h) CAC, $F_{1}$, E = 0.838, (i) CAC, $F_{2}$, E = 0.839.

To further explore the visual‐temporal patterns among subjects without DM, with DM, and with CAC, Figure 2 goes beyond numerical representations. It not only displays the coefficients of PARAFAC decomposition factors $F_{1}$ and $F_{2}$ extracted from the 3‐way array models but also vividly illustrates their corresponding FRPs as grayscale images of texture and spatial entropy. This additional layer of detail enhances the understanding of temporal dynamics within the data.

**Figure 3:**
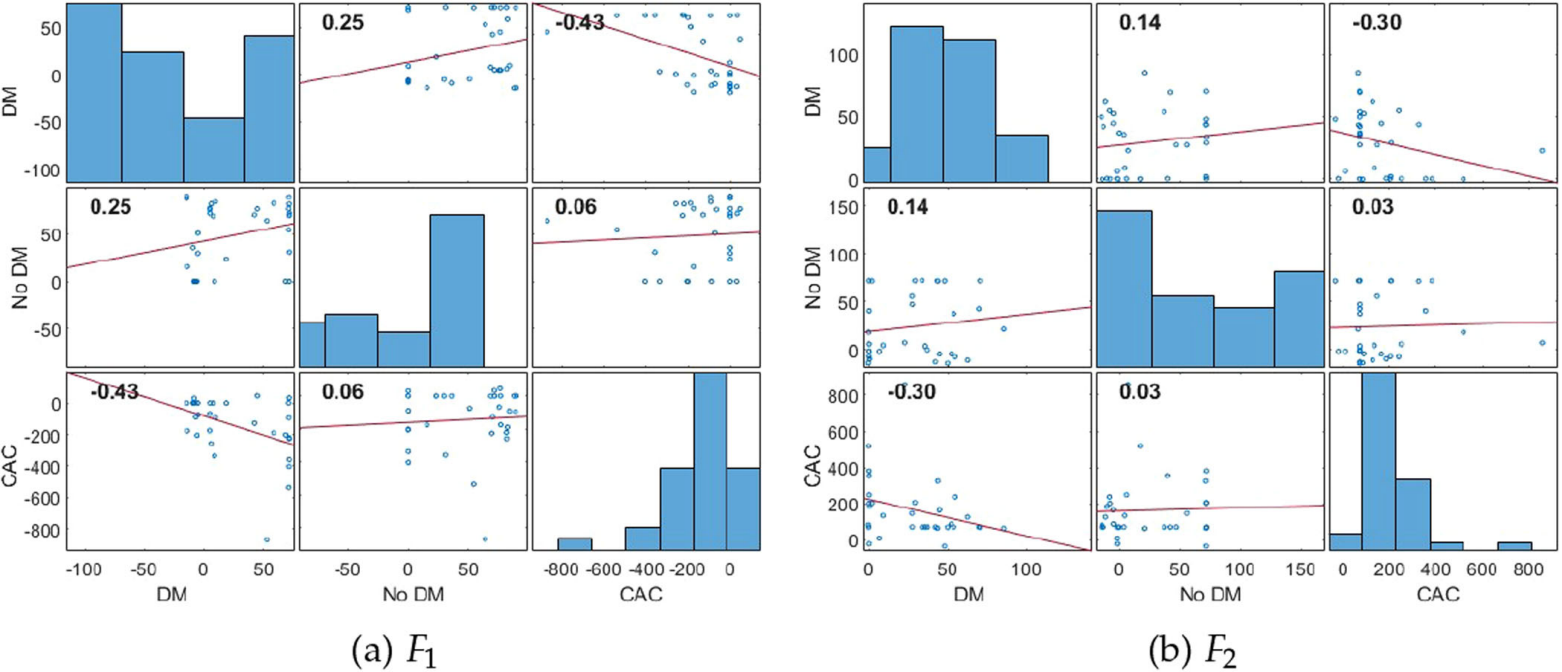
Pearson's correlation coefficients between all pairs of DM, no DM, and CAC tertiles 2 & 3 in terms of PARAFAC decomposition factors $F_{1}$ and $F_{2}$; where each diagonal subplot contains the coefficient distribution as a histogram. (a) $F_{1}$, (b) $F_{2}$.

Moreover, as depicted in Figure 3, the intricacies of relationships are discovered through Pearson correlation coefficients of the two PARAFAC decomposition factors. The figure exhibits subplots portraying Pearson correlation coefficients among all pairs involving DM, no DM, and CAC (tertiles 2 and 3). Within each diagonal subplot as shown in Figure 3, the distribution of the variable is presented as a histogram. The plots, along with their corresponding correlation coefficients, underscore that both tensor decomposition factors $F_{1}$ and $F_{2}$ of diabetes exhibit stronger correlations with CAC compared to the counterparts without diabetes and CAC. This observation emphasizes the distinctive and more interconnected relationship between the diabetes category and coronary artery calcification.

## 4 Discussions

In the domain of data analysis, the utilization of patient characteristics in the 2‐way array analysis, coupled with a k‐NN search, has unveiled a relationship–”patients with diabetes demonstrate a connection to those falling within CAC tertiles 2 and 3. However, intriguingly, this correlation is not reciprocated; the reverse relationship does not hold true. This preliminary exploration underscores the intricacies of the interplay between diabetes and CAC and suggests a deeper investigation.

To explore a more comprehensive understanding, the analytical scope is expanded through the inclusion of both patient characteristics and dental health in the 3‐way array analysis. This augmented approach is further enhanced by a k‐NN search, shedding light on the bidirectional relationship between patients with diabetes and those falling within CAC tertiles 2 and 3. By encompassing not only patient characteristics but also the subtle dimension of dental health, this analysis promises a more nuanced depiction of the complex dynamics at play.

The 3‐way array analysis, integrating patient characteristics, dental health, and their interaction, has the potential to reveal hidden patterns and dependencies that might have remained undiscovered in a simpler 2‐way analysis. The bidirectional relationship between diabetes and CAC tertiles 2 and 3 is anticipated to emerge with greater clarity, offering valuable insights into how these factors interact and influence each other within the data set.

The implications drawn from the visual representation of the FRPs are intriguing. While a relationship is shared by patients with diabetes and those falling within CAC tertiles 2 and 3, Figure 2 reveals that each cohort—patients without diabetes, those with diabetes, and those in CAC tertiles 2 and 3—displays distinctive nonlinear dynamic and textural patterns. This diversity in patterns becomes a focal point for analysis and understanding.

To quantify these unique patterns, a method for computing the entropy of an FRP is employed. Examining the FRPs of each cohort unveils distinct characteristics. In the nondiabetic group, the FRP showcases strong recurrence in the upper‐left image region, accompanied by intermediate texture in other regions. The FRP of the CAC group, in contrast, exhibits the most even distribution of recurrence and texture throughout the image. Meanwhile, the diabetic cohort' s FRP reveals distributions of strong recurrence in both diagonal and off‐diagonal orientations.

The quantitative expression of these unique differences among the three cohorts is facilitated by the fuzzy entropy of the FRPs. This measurement serves as a valuable metric, offering a subtle and detailed understanding of the complex relationships within the data. Importantly, fuzzy entropy values provide a means to identify the most influential health condition within the broader context of overall health, diabetes, and cardiovascular risk for an individual patient.

Exploring the bidirectional relationship between diabetes and cardiovascular risk, particularly concerning CAC, holds several implications for public health, clinical management, and preventive strategies. The complex interplay between diabetes and CAC underscores the need for understanding this relationship due to several key reasons. Individuals with diabetes face an elevated risk of cardiovascular diseases, including coronary artery disease [32] Diabetes contributes to endothelial dysfunction, inflammation, and metabolic disturbances, accelerating atherosclerosis‐”the primary factor leading to CAC [33].

CAC serves as a surrogate marker for atherosclerosis, reflecting the cumulative burden of atherosclerotic plaque and correlating with an increased risk of adverse cardiovascular events [34]. Understanding the bidirectional relationship aids in deciphering whether diabetes directly accelerates CAC or if CAC contributes to the progression of diabetes‐related cardiovascular complications. This insight allows for better risk stratification and prediction models. By identifying individuals with diabetes at a higher risk of developing CAC, healthcare providers can tailor interventions and preventive measures more effectively, informing personalized treatment plans and lifestyle modifications.

Discovering the bidirectional relationship informs the development of targeted interventions. For instance, if diabetes significantly contributes to CAC, aggressive diabetes management becomes a preventive strategy. Conversely, if CAC drives diabetes‐related cardiovascular risk, interventions targeting atherosclerosis progression may take precedence. Diabetes is a global health concern [1], and its association with cardiovascular diseases poses a substantial public health burden [2]. Investigating the bidirectional relationship sheds light on potential avenues for population‐wide interventions, such as screening programs and lifestyle interventions, to mitigate the impact of diabetes on CAC and subsequent cardiovascular events.

Furthermore, understanding the bidirectional relationship between diabetes and cardiovascular risk, especially in the context of CAC, offers several advantages over examining a non‐bidirectional relationship. It enables researchers and clinicians to gain a more comprehensive understanding of the complex interplay between diabetes and CAC, considering how each condition influences and exacerbates the other over time. Bidirectional relationship analysis enhances risk stratification models, recognizing the reciprocal influence between diabetes and CAC for a more accurate assessment of an individual' s overall cardiovascular risk. This approach contributes to better‐informed decision‐making regarding treatment and preventive strategies.

This study introduced a novel application of tensor decomposition and fuzzy recurrence plots to examine the complex interrelationship between stomatognathic diseases, DM, and cardiovascular risk–focusing particularly on the bidirectional link between DM and cardiovascular risk. The multi‐dimensional analytical approach enabled a comprehensive characterization of patterns that are often overlooked in conventional statistical methods. The findings revealed consistent associations between dental conditions, such as periodontal disease, and markers of metabolic dysfunction and cardiovascular risk, aligning with existing evidence of shared inflammatory and vascular pathways.

While the retrospective cross‐sectional design limits causal inference, the observed patterns highlight the potential role of oral pathology as a clinical indicator within systemic disease networks. Future work may benefit from mediation analyses to formally assess whether oral conditions mediate the bidirectional relationship between diabetes and cardiovascular risk. Such approaches could elucidate causal pathways and clarify whether improving oral health may indirectly influence cardiometabolic outcomes.

Limitations of the present study include reliance on electronic health records with possible underreporting or variability in diagnostic criteria, as well as the absence of behavioral or socioeconomic variables that may confound observed associations. Nevertheless, the use of multi‐way array modeling offers a scalable framework for integrating complex, multimodal health data.

## 5 Conclusion

This study identified distinct multidimensional patterns linking stomatognathic diseases, DM, and cardiovascular risk by applying multi‐way array decomposition and fuzzy recurrence analysis. The results demonstrate a bidirectional association between DM and CAC scores in the context of oral conditions, suggesting interconnected disease processes. These findings support the hypothesis that oral health reflects broader systemic disturbances and may serve as a proxy marker for cardiometabolic risk.

While the cross‐sectional nature of the data limits causal inference, the analytical approach reveals patterns that merit further investigation. The inclusion of oral health in systemic disease frameworks may enhance early identification of at‐risk individuals. Future longitudinal studies, including mediation analyses, could help determine whether improving oral health contributes to cardiometabolic risk reduction through indirect pathways.

## Author Contributions

**Tuan D Pham:** Conceptualization; investigation; writing – original draft; methodology; validation; visualization; software; formal analysis; writing – review and editing.

## Ethics Statement

The data are anonymized and publicly available, so ethical approval is not required for this study.

## Conflicts of Interest

The authors declare no conflicts of interest.

## Data Availability

The data that support the findings of this study are openly available in Figshare at https://figshare.com/articles/dataset/S1Data/13391239file=25796773, reference number https://doi.org/10.1371/journal.pone.0243232.s001.
